# Artificial intelligence-assisted diagnosis and histopathological grading of bladder cancer: current status, challenges, and future directions

**DOI:** 10.3389/fdgth.2026.1708289

**Published:** 2026-02-05

**Authors:** Lihao Zhang, Yinghao Zhong, Gang Yang, Lige Huang, Aijia Deng, Mengxin Ao, Jiabing Li

**Affiliations:** 1 North Sichuan Medical College, Nanchong, China; 2 Department of Urology, Mianyang Central Hospital, Mianyang, China; 3 Southwest Medical University, Luzhou, China; 4 Mianyang Maternal and Child Health Hospital, Mianyang, China; 5 Department of Urology, Sichuan Mianyang 404 Hospital, Mianyang, China

**Keywords:** artificial intelligence, bladder cancer, deep learning, digital pathology, histopathological grading, precision medicine

## Abstract

Bladder cancer is one of the most prevalent malignant tumors of the urinary system worldwide, and its diagnosis and histopathological grading are crucial for clinical decision-making and prognostic evaluation. Although traditional methods such as cystoscopy, imaging, and histological examination remain the clinical gold standard, they suffer from significant subjectivity and interobserver variability. Artificial intelligence (AI), particularly deep learning (DL)–based approaches, has demonstrated substantial potential in image recognition, histopathological grading, and risk prediction. This review systematically summarizes recent advances in the application of AI to bladder cancer diagnosis and grading, covering imaging analysis, digital pathology, molecular marker identification, and AI-driven clinical decision support. In addition, key challenges associated with current AI technologies are discussed, including data quality, model generalizability, interpretability, clinical translation, and ethical and regulatory considerations. Finally, future research directions are outlined, including multimodal AI integration, incorporation of biomarkers, and the development of intelligent decision-support systems. Overall, AI is poised to play an increasingly important role in improving diagnostic accuracy and enabling personalized management of bladder cancer, thereby advancing the intelligent and data-driven management of urologic oncology.

## 1 Epidemiology and pathological foundations of bladder cancer

### 1.1 Epidemiological characteristics of bladder cancer

Bladder cancer is one of the most common malignant tumors of the urinary system worldwide, ranking among the top ten most frequently diagnosed cancers globally. Its incidence varies significantly across regions and has shown a steady increase trend in countries such as China in recent years. According to relevant studies, the incidence of bladder cancer is substantially higher in males than in females, with the majority of cases occurring in middle-aged and elderly populations, particularly in men over 50 years old (1). Notably, the high recurrence rate and progression of bladder cancer pose significant challenges to clinical management, necessitating long-term follow-up to monitor disease recurrence and progression (2). The pathogenesis of bladder cancer is complex and involves both environmental and genetic factors. Cigarette smoking represents the most important risk factor, accounting for over 50% of cases. Additionally, occupational exposure to aromatic amines, chronic cystitis, bladder stones, and a history of pelvic radiation therapy are all strongly associated with the development of bladder cancer (3). Epidemiological studies have shown that the incidence and mortality rates of bladder cancer differ among countries and regions, largely influenced by environmental exposure, lifestyle factors, and disparities in healthcare resources (4). Diagnosis of bladder cancer primarily relies on cystoscopic examination and histopathological evaluation. However, conventional methods such as white-light cystoscopy have limitations, particularly in detecting flat lesions and small foci. In recent years, advanced optical imaging technologies—such as blue-light cystoscopy and fluorescence cystoscopy—have substantially improved tumor detection rates and diagnostic accuracy (5). Moreover, the integration of AI, particularly DL algorithms, into imaging and pathological analysis has enhanced early detection and risk stratification, supporting more personalized treatment decisions (1). Epidemiological studies also underscore the importance of early detection and accurate diagnosis. Given the high recurrence and progression risks associated with bladder cancer, early diagnosis and precise grading are crucial for formulating appropriate treatment strategies and improving patient outcomes. With the advancement of AI technologies, multimodal analytical approaches integrating clinical, imaging, and pathological data offer promising prospects for achieving precise diagnosis and dynamic risk assessment, thus advancing the field of precision medicine (4).

### 1.2 Major pathological types and grading standards of bladder cancer

Bladder cancer exhibits a variety of pathological types, with urothelial carcinoma (also known as transitional cell carcinoma) being the most common, accounting for more than 90% of all cases. Less frequent pathological types include squamous cell carcinoma, adenocarcinoma, and small cell carcinoma (1). Based on tumor invasion depth and histological differentiation, urothelial carcinoma is classified into non–muscle-invasive bladder cancer (NMIBC) and muscle-invasive bladder cancer (MIBC), a distinction that is critical to therapeutic decision-making and prognostic evaluation (6). The histopathological grading of bladder cancer is primarily based on the degree of cellular differentiation and morphological characteristics. Traditional grading systems include the World Health Organization (WHO) classification and the International Society of Urological Pathology (ISUP) system, which generally categorize tumors into low-grade (G1), intermediate-grade (G2), and high-grade (G3) lesions. Low-grade tumors exhibit well-differentiated cells, slow growth, and favorable prognosis, while high-grade tumors are poorly differentiated, highly invasive, and associated with worse outcomes (7). In recent years, advances in molecular pathology have promoted the development of molecular subtyping of bladder cancer. Gene expression profiling has led to the identification of several molecular subtypes, primarily including the “basal” and “luminal” subtypes. Basal-type bladder cancers are typically high-grade, muscle-invasive, and responsive to neoadjuvant chemotherapy, whereas luminal-type tumors are usually non–muscle-invasive with relatively better prognosis but may be more responsive to immune checkpoint inhibitors (8). These molecular subtypes provide an important biological and clinical framework for personalized therapy in bladder cancer. Tumor staging is also essential in pathological evaluation. The TNM staging system is the internationally accepted standard: T stage indicates the depth of tumor invasion, N stage denotes lymph node involvement, and M stage refers to distant metastasis. Accurate staging is critical for therapeutic planning and prognostic assessment (9). However, traditional histopathological grading and staging suffer from subjectivity and interobserver variability. DL-driven AI-assisted digital pathology, enables automated analysis and grading of histopathological images, thereby improving diagnostic objectivity and consistency (10). Moreover, pathological diagnosis of bladder cancer involves identifying rare histologic variants such as adenoid cystic carcinoma and micropapillary carcinoma, which have distinct clinical behaviors and prognoses. AI has shown promise in recognizing and classifying these rare variants, thereby assisting pathologists in improving diagnostic accuracy (1). In summary, urothelial carcinoma is the predominant pathological type of bladder cancer. Grading systems include traditional histological classifications and emerging molecular subtyping. With continued advances in AI technologies, digital pathology and deep learning–based diagnostic tools are expected to further enhance grading accuracy and support more precise and personalized clinical management of bladder cancer.

## 2 Methods

### 2.1 Literature search strategy

This narrative review aims to summarize recent advances in artificial intelligence–assisted diagnosis and histopathological grading of bladder cancer. A comprehensive literature search was performed in major biomedical databases, including PubMed, Web of Science, and Scopus, covering studies published up to September 2025.

The search strategy combined Medical Subject Headings terms and free-text keywords related to bladder cancer and artificial intelligence, including, but not limited to “bladder cancer,” “urothelial carcinoma,” “artificial intelligence,” “machine learning,” “deep learning,” “radiomics,” “digital pathology,” “cystoscopy,” and “histopathological grading.”

Eligible articles included original research studies, review articles, and clinical investigations focusing on AI applications in bladder cancer diagnosis, grading, staging, or clinical decision support. Studies unrelated to bladder cancer, non-AI–based approaches, conference abstracts without full text, and non-English publications were excluded.

Relevant articles were initially screened based on titles and abstracts, followed by full-text evaluation to assess relevance. Additional studies were identified by manually reviewing the reference lists of key publications. The final selection of articles was based on methodological quality, scientific relevance, and clinical significance.

### 2.2 Review methodology

For clarity and consistency, the term AI is used as an overarching concept, encompassing machine learning (ML) and DL, with DL representing a subset of ML. Throughout this review, ML and DL are specified explicitly when referring to particular algorithmic approaches. Similarly, histopathological grading is used to denote grading based on microscopic pathological assessment, whereas tumor grading is used as a broader clinical term encompassing pathological and clinical considerations.

## 3 Traditional diagnostic and grading approaches for bladder cancer and their limitations

### 3.1 Imaging, endoscopy, and histological diagnosis

Traditional diagnosis of bladder cancer primarily relies on imaging examinations, endoscopic procedures, and histopathological analysis. In terms of imaging, computed tomography (CT) and magnetic resonance imaging (MRI) are commonly used adjunctive diagnostic tools. CT urography serves as a primary method for initial screening and assessment of bladder cancer, while multiparametric MRI demonstrates high accuracy in local staging. In particular, diffusion-weighted imaging (DWI) provides valuable insights into tumor physiology and the depth of invasion (2, 6). In addition, functional imaging modalities such as dynamic contrast-enhanced MRI (DCE-MRI) and positron emission tomography-computed tomography (PET-CT), although still under development, offer more comprehensive information on tumor biology and contribute to individualized treatment planning (11). Cystoscopy, especially white-light cystoscopy, remains the gold standard for the diagnosis of bladder cancer. However, its sensitivity is limited in detecting flat lesions such as carcinoma *in situ* (CIS). To address this limitation, advanced techniques such as photodynamic diagnosis (PDD) using fluorescence cystoscopy and narrow-band imaging (NBI) have been introduced into clinical practice. These modalities significantly enhance tumor detection rates, improve the completeness of tumor resection, and reduce residual disease and recurrence rates (12–14). Furthermore, emerging technologies such as confocal laser endomicroscopy and optical coherence tomography show promise in the grading and staging of bladder lesions, although robust prospective validation is still lacking, though clinical validation is still limited and further studies are needed (14). Histopathological diagnosis relies on tissue samples obtained through transurethral resection of bladder tumor (TURBT). Tumor confirmation and grading are performed based on microscopic morphological evaluation and immunohistochemical staining. Histological examination forms the foundation for determining tumor stage and grade, which directly influences treatment decisions. Despite its central role, conventional histopathology is constrained by interobserver variability and limited capacity to capture intratumoral heterogeneity, thereby compromising diagnostic consistency and accuracy (1, 15).

### 3.2 Challenges in conventional histopathological grading systems

The histopathological grading of bladder cancer has traditionally followed the standards established by the World Health Organization (WHO) and International Society of Urological Pathology (ISUP), classifying tumors into low-grade (G1), high-grade (G3), and intermediate-grade (G2) categories (7). This grading system is primarily based on morphological features of tumor cells, such as nuclear size, nuclear pleomorphism, nucleolar prominence, and mitotic activity. The purpose of grading is to evaluate tumor aggressiveness and prognostic risk, thereby guiding clinical treatment decisions. However, conventional grading systems face several challenges. First, G2 tumors exhibit considerable morphological heterogeneity, resulting in variability in prognosis and treatment response. This heterogeneity complicates the development of standardized therapeutic strategies for intermediate-grade tumors (7). These challenges are further compounded by the inherent subjectivity of conventional grading, as discussed in the following section (15). Moreover, traditional grading systems fall short in capturing the molecular heterogeneity and dynamic biological behavior of tumors, limiting risk stratification and therapeutic personalization and biological behavior of tumors, thereby limiting their utility in precision medicine (16). In recent years, molecular subtyping and gene expression profiling have provided new perspectives for grading and prognostic evaluation of bladder cancer. Classification based on molecular characteristics has revealed the biological diversity of bladder cancer, identifying distinct pathogenic mechanisms and clinical behaviors, and laying the groundwork for personalized treatment (17, 18). Nonetheless, widespread clinical implementation remains limited by high costs, technical complexity, and restricted accessibility.

### 3.3 Subjectivity and consistency issues in diagnosis and grading

Subjectivity remains a significant issue in the diagnosis and histopathological grading of bladder cancer. Traditional histopathological assessment relies on the visual evaluation of tissue sections by pathologists, and is influenced by individual experience, training background, and workload. This leads to considerable interobserver variability in diagnosis (7, 15). Such subjectivity not only compromises diagnostic accuracy but also impacts treatment decisions and prognostic evaluation. Moreover, bladder cancer exhibits pronounced histological heterogeneity. This issue is particularly pronounced in intermediate-grade tumors (7). Urinary cytology, as an adjunctive diagnostic tool, demonstrates high specificity for high-grade tumors but low sensitivity for low-grade tumors, and its interpretation also suffers from subjectivity (19). These limitations reduce the clinical effectiveness of conventional diagnostic approaches and have prompted the exploration of more objective and standardized diagnostic tools. The emergence of digital pathology and AI technologies offers new opportunities to address issues of subjectivity and consistency. Deep learning algorithms applied to digitized histopathological slides enable automated tumor detection, grading, and staging, improving diagnostic accuracy and reproducibility under controlled experimental conditions (1, 20). However, AI-assisted diagnosis still faces challenges such as data standardization, model interpretability, and clinical validation, and has yet to fully replace traditional pathological evaluation (21). In summary, traditional methods for diagnosing and grading bladder cancer remain central in clinical practice, but their inherent subjectivity and variability limit diagnostic reliability. Future diagnostic paradigms integrating molecular information with AI-driven analytical frameworks are expected to further improve accuracy and support the goals of precision medicine.

## 4 Current applications of artificial intelligence in the diagnosis and grading of bladder cancer

### 4.1 Technical principles and methods of AI-assisted diagnosis

The application of AI in the diagnosis of bladder cancer primarily relies on machine learning (ML) and DL technologies. Machine learning algorithms enable the automated extraction of informative features from large-scale datasets, facilitating classification and prediction tasks. Deep learning, particularly convolutional neural networks (CNNs), demonstrates exceptional performance in image recognition and processing tasks. Typical AI systems consist of several steps, including data preprocessing, feature extraction, model training, and validation. Through these processes, AI systems can automatically identify tumor regions and extract grading and staging information from complex medical images and digitized histopathological slides, thereby reducing human subjectivity and enhancing diagnostic objectivity and consistency. In bladder cancer diagnosis, AI is evolving from reliance on single data modalities to the integration of multimodal data, combining imaging, pathology, and molecular biology to provide more comprehensive diagnostic support. These developments highlight AI's potential to significantly enhance diagnostic accuracy and efficiency (1).

### 4.2 Applications of AI in imaging and endoscopic analysis

Traditionally, the imaging diagnosis of bladder cancer has relied on modalities such as computed tomography (CT), magnetic resonance imaging (MRI), and cystoscopy. However, these methods often suffer from subjectivity and poor diagnostic consistency. In recent years, AI technologies have demonstrated increasing maturity in bladder cancer imaging, particularly in cystoscopic image analysis and radiomics. Deep learning-based DL models can automatically identify tumor lesions during cystoscopy, significantly improving the identification of flat and small lesions. For instance, an AI classification platform using blue-light cystoscopy images demonstrated a sensitivity of 95.77% and a specificity of 87.84% for detecting malignant lesions. The average sensitivity and specificity for identifying tumor invasiveness reached 88% and 96.56%, respectively, substantially outperforming conventional manual diagnosis (5). Additionally, AI-assisted radiomics techniques can extract high-dimensional features from CT and MRI scans, enabling precise tumor staging and grading, and supporting the development of individualized treatment strategies (22). In endoscopic analysis, AI combined with enhanced imaging techniques—such as narrow-band imaging and optical coherence tomography—can provide real-time assistance to physicians in identifying bladder cancer lesions, thereby reducing missed diagnoses and misdiagnoses. Although some of these technologies are still undergoing clinical validation, studies have already shown that AI-assisted endoscopic diagnosis holds promise for improving the early detection rate and diagnostic accuracy of bladder cancer (13).

### 4.3 Applications of AI in histopathological image analysis

Histopathology remains the gold standard for diagnosing bladder cancer, but traditional pathological diagnosis heavily relies on the experience of pathologists and is often limited by subjectivity and poor reproducibility. With the advancement of digital pathology, AI-based histopathological image analysis has become increasingly prevalent. In particular, deep learning-based image recognition algorithms can automatically detect, segment, classify, and grade tumor tissues. DL models trained on digitized whole slide images (WSIs) are capable of identifying various histological subtypes and grades of bladder cancer, assisting pathologists in improving diagnostic efficiency and accuracy. For example, a deep learning model combining multiple instance learning (MIL) with attention mechanisms achieved an excellent F1 score of 0.85 in bladder cancer histopathological grading. Importantly, the attention maps demonstrated strong spatial correspondence with high-grade tumor regions, enhancing model interpretability (23). Moreover, AI can uncover subvisual features in pathological images that are difficult for the human eye to detect, enabling the prediction of tumor molecular characteristics and prognostic information, thus supporting precision medicine. Overall, AI-assisted pathological analysis not only reduces pathologist workload but also promotes greater standardization and objectivity in histopathological diagnosis (21, 24).

### 4.4 Enhancing consistency in histopathological grading with AI

Histopathological grading of bladder cancer is a critical determinant for guiding clinical treatment and prognosis. Given the well-recognized limitations of conventional grading, AI-assisted systems have been proposed to enhance grading consistency. This is particularly relevant for intermediate-grade tumors, where reproducibility remains limited. AI-assisted systems, trained on large annotated datasets, can provide stable and reproducible grading outcomes, helping to standardize diagnostic criteria. For instance, AI-assisted histopathological grading reduces subjectivity and improves the accuracy of identifying high-grade tumors, thereby supporting more informed treatment decisions (25). Moreover, AI-supported grading systems have demonstrated potential in clinical practice to improve prognostic assessment by more accurately predicting tumor progression risk and patient survival, thus facilitating the development of personalized treatment strategies. With ongoing advancements in digital pathology and AI, these technologies are expected to play an increasingly vital role in enhancing the consistency and quality of bladder cancer grading (10, 20). To facilitate comparison of representative studies across different imaging modalities and AI tasks, we summarize key research findings in Table 1.

**Table 1:** Summary of representative artificial intelligence studies in bladder cancer categorized by data modality, clinical task, model architecture, and reported performance metrics.

Modality	Task	Reference	Model/method	Data type/scale	Reported performance
Cystoscopy (Blue-light)	Tumor detection/classification	Ali et al. (5)	CNN-based deep learning classifier	Blue-light cystoscopy images during TURBT	Sensitivity 95.77%, Specificity 87.84%
Cystoscopy (Enhanced imaging)	Lesion detection (review)	Shkolyar et al. (13)	CNN-based AI systems (review)	WL + enhanced cystoscopy images	NR
CT/MRI	Tumor staging	Sarkar et al. (22)	Hybrid deep–machine learning model	CT imaging data	Accuracy ∼85%, AUC NR
CT/MRI	Imaging-based staging (review)	Wong et al. (6)	Radiomics + ML (review)	CT/mpMRI	NR
Histopathology (WSI)	T-stage prediction	Bera et al. (9)	ML-based image analysis	Digital pathology WSIs	AUC NR
Histopathology (WSI)	histopathological grading	Fuster et al. (23)	Weakly supervised DL (MIL)	WSIs (NMIBC)	F1-score ≈ 0.85
Histopathology (WSI)	Tumor classification (review)	Jiang et al. (10)	CNN/DL (review)	Digital pathology images	NR
Histopathology (WSI)	AI in pathology (review)	Marra et al. (21)	CNN, MIL, transformer (review)	WSIs across cancers	NR
Multimodal (WSI + Genomics)	Risk stratification (conceptual)	Yang et al. (1)	Multimodal DL (review)	Imaging + molecular data	NR
Non-imaging (Blood & urine droplets)	Cancer detection	Demir et al. (26)	AI-based pattern recognition	Blood & urine samples	Accuracy >90%

CNN, convolutional neural network; WSI, whole-slide image; TURBT, transurethral resection of bladder tumor; MIL, multiple instance learning; NR, not reported.

## 5 Challenges and unmet clinical needs in AI applications

### 5.1 Data quality and inter-center heterogeneity

Despite the promising performance demonstrated by artificial intelligence in bladder cancer diagnosis and grading, the quality and representativeness of training data remain fundamental limitations. Most existing DL models are developed using retrospective datasets derived from single institutions or limited geographic regions. Such datasets often reflect institution-specific imaging protocols, pathological processing methods, and patient demographics, resulting in substantial data heterogeneity.

This heterogeneity is not merely a technical inconvenience but a major source of bias, as models trained under these conditions may inadvertently learn site-specific patterns rather than true disease-related features, thereby inflating apparent model performance. Consequently, reported high accuracy in internal validation settings may be substantially overestimated, limiting the generalizability of these models when applied to external cohorts or real-world clinical environments.

### 5.2 Limited generalizability and external validation

A critical shortcoming of many current AI studies is the lack of rigorous external and prospective validation frameworks. While internal cross-validation is commonly reported, relatively few studies evaluate model performance across independent multicenter datasets. As a result, the robustness of DL models under real-world clinical variability remains largely unproven.

Importantly, limited generalizability has direct clinical consequences. AI systems that perform inconsistently across institutions may generate unreliable predictions, potentially undermining clinician confidence and increasing the risk of inappropriate diagnostic or therapeutic decisions. Without standardized validation frameworks and transparent reporting of failure modes, premature clinical deployment of DL models may fail to enhance patient care and, in some cases, introduce clinical risk.

### 5.3 Model generalizability and interpretability

The generalizability and interpretability of AI algorithms for bladder cancer diagnosis and grading remain central challenges for their clinical application. Generalizability refers to a model's ability to perform well on previously unseen data, while interpretability pertains to the transparency and trustworthiness of the model's decision-making process. Many DL models demonstrate strong performance in controlled experimental settings but exhibit marked performance degradation when applied across institutions or devices, indicating limited robustness. Xiangxiang et al. noted that, based on currently available evidence, while AI-based bladder cancer diagnostics relying on deep learning have improved diagnostic efficiency and consistency, their robustness and reliability in multi-institutional and multi-device environments remain limited (1). Similarly, research by Ali et al. demonstrated that, although AI achieved high sensitivity and specificity in classifying blue light cystoscopy images, small sample sizes and data heterogeneity constrained the model's broader applicability (5). Regarding interpretability, DL models—especially deep neural networks—are often regarded as “black boxes,” with opaque internal mechanisms that hinder understanding of how decisions are made. Jiang et al. reviewed AI applications in oncologic pathology and highlighted the lack of interpretability as a major barrier to clinician trust and adoption (10). Kiani et al. investigated AI-assisted histopathologic diagnosis in liver cancer, providing important cautionary insights applicable to bladder cancer pathology and found that while accurate AI predictions improved pathologists' performance, erroneous predictions could mislead diagnoses, underscoring the risks associated with non-interpretable systems (27). To address these challenges, current research has explored the incorporation of attention mechanisms and MIL to improve local interpretability. For example, Fuster et al. proposed a bladder cancer grading model where regional attention scores contributed to explainability (23). In addition, hybrid models combining traditional feature engineering with deep learning are being developed to enhance both transparency and model stability. Improving generalizability will require large-scale, multicenter training and rigorous external validation, while interpretability demands transparent model architectures and clinician-oriented visualization tools.

### 5.4 Clinical validation and translational barriers

Although AI has made significant technical progress in the diagnosis and grading of bladder cancer, its clinical validation and real-world translation still face numerous obstacles. Most existing DL models remain at the research stage and lack support from large-scale, multi-center, prospective clinical trials, limiting their widespread adoption in clinical practice. Ma et al. noted that AI applications in bladder cancer have not been fully integrated into clinical workflows, largely due to insufficient clinical validation and a lack of algorithmic transparency (4). Shkolyar et al. emphasized that while enhanced cystoscopic technologies and AI-assisted systems show promise in laboratory and small-scale clinical settings, their impact on tumor resection quality and patient outcomes has yet to be confirmed by large clinical trials (13). Furthermore, Gao et al.'s systematic review of AI in gastrointestinal tumor diagnosis highlighted the lack of standardized data protocols and large-scale validation as major barriers to clinical translation (28). Another significant challenge in clinical implementation is the difficulty of integrating AI systems into existing medical workflows. Hoskin et al. pointed out that effective AI deployment in cancer care requires alignment with clinical protocols, along with appropriate training for healthcare professionals to understand and operate AI tools. The level of trust and acceptance from clinicians also plays a crucial role in AI adoption, particularly when incorrect AI predictions may lead to clinical risk. In addition, regulatory approval and the establishment of standards represent critical bottlenecks. Bera et al. stressed that the application of AI in digital pathology must undergo rigorous validation and regulatory scrutiny to ensure safety and effectiveness, yet globally harmonized regulatory frameworks are currently lacking. Verlingue et al. further emphasized that with the emergence of large language models and other novel AI technologies, clinical evaluation and bias assessment have become increasingly important and must be addressed through prospective global clinical trials. Overall, successful clinical translation will depend on prospective validation, workflow integration, and regulatory alignment.

### 5.5 Ethical, privacy, and regulatory concerns

Beyond technical limitations, the clinical deployment of AI in bladder cancer diagnosis raises significant ethical, privacy, and regulatory concerns. The reliance of AI systems on large-scale patient data introduces inherent tensions between data sharing and privacy protection. Inadequate governance mechanisms may expose patients to risks related to data misuse, re-identification, or unauthorized access.

Furthermore, algorithmic bias arising from imbalanced datasets may exacerbate healthcare disparities, particularly when AI systems are deployed across diverse populations. From a regulatory perspective, the absence of unified international standards for AI-based medical devices complicates clinical translation. Without clear requirements regarding data quality, model transparency, and post-market surveillance, regulatory uncertainty remains a major bottleneck to widespread adoption.

Therefore, addressing these challenges requires coordinated efforts encompassing robust data governance frameworks, explainable AI development, clinician training, and harmonized regulatory pathways. The major technical and regulatory challenges associated with AI-assisted bladder cancer diagnosis, along with proposed mitigation strategies, are summarized in Table 2.

**Table 2:** Major challenges in artificial intelligence applications for bladder cancer diagnosis and histopathological grading, and proposed mitigation strategies.

Challenge	Description	Proposed mitigation strategy	Key references
Limited dataset size	Most AI studies rely on single-center, small cohorts	Multicenter data sharing, federated learning	Ma et al. (4); Gao et al. (28)
Interobserver variability	histopathological grading varies among experts	Consensus annotation, AI-assisted standardization	Tosoni et al. (25); Försch et al. (15)
Poor model generalizability	Performance drops across scanners and centers	External validation, domain adaptation	Yang et al. (1); Zhou et al. (29)
Lack of interpretability	“Black-box” DL limits clinician trust	Explainable DL (attention maps, heatmaps)	Jiang et al. (10); Marra et al. (21)
Integration into clinical workflow	AI tools not aligned with real-world practice	Workflow-aware system design and clinician training	Hoskin (30)
Regulatory & ethical issues	Data privacy, bias, and accountability concerns	Robust governance frameworks and validation trials	Lai et al. (31); Zhang et al. (32)

## 6 Future directions for AI in bladder cancer diagnosis and grading

### 6.1 The potential of multimodal AI systems

**Figure 1:**
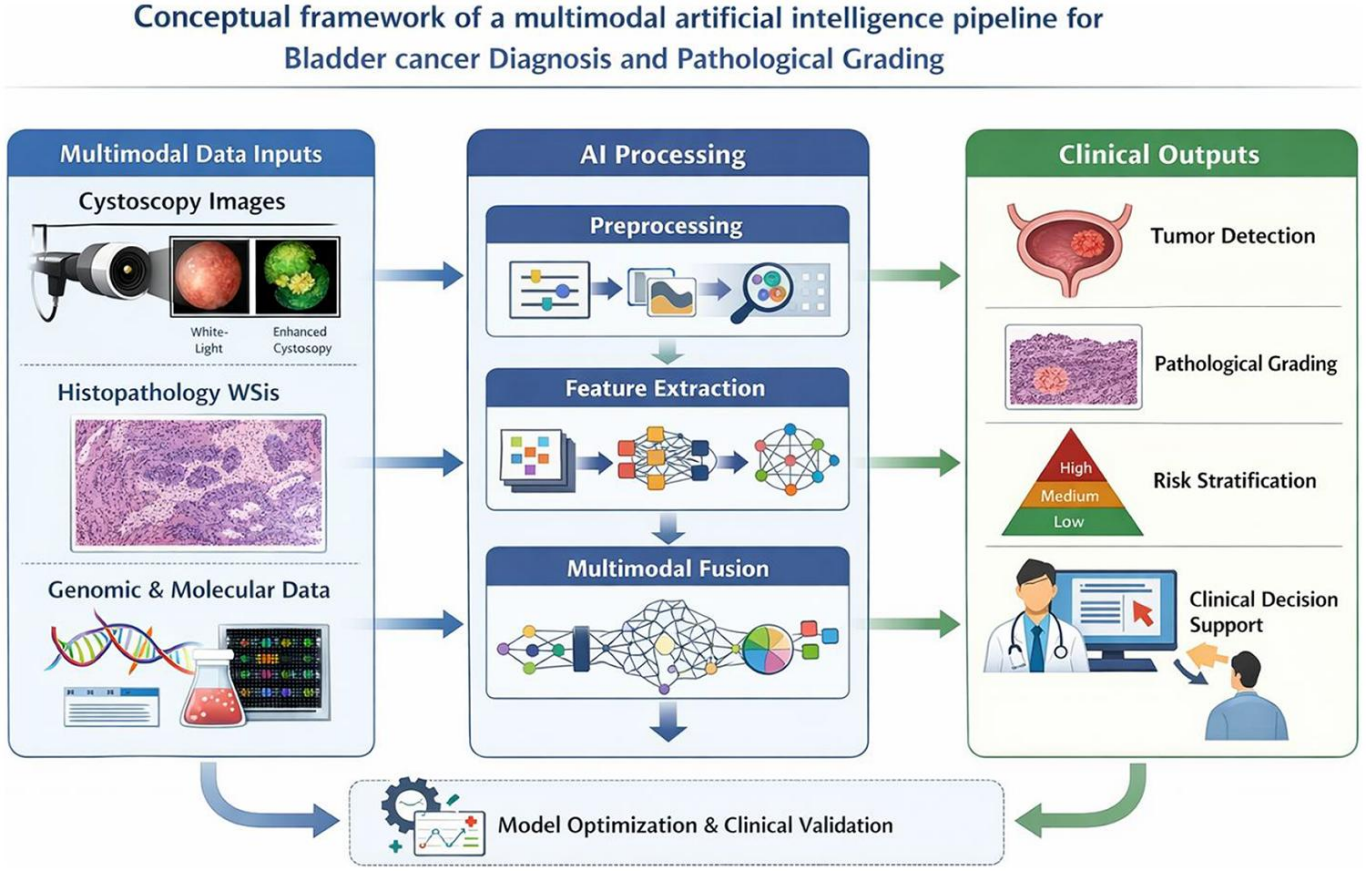
A conceptual illustration of a multimodal AI pipeline for bladder cancer diagnosis and histopathological grading. Multisource inputs, including cystoscopic imaging (white-light and enhanced cystoscopy), digital histopathology whole-slide images (WSIs), and genomic or molecular data, are processed through AI-driven preprocessing, feature extraction, and multimodal fusion modules. The integrated AI system generates clinically relevant outputs such as tumor detection, histopathological grading, molecular subtype inference, risk stratification, and decision support for personalized treatment planning. This framework highlights the potential of multimodal AI to improve diagnostic accuracy, consistency, and precision in bladder cancer management.

Multimodal AI systems, which integrate diverse data sources such as imaging, pathology, molecular biology, and clinical information, demonstrate substantial potential in the diagnosis and grading of bladder cancer. The pronounced heterogeneity and complex biological characteristics of bladder cancer make it difficult for single-modality data to comprehensively capture disease status. In recent years, advances in deep learning have enabled effective fusion and joint analysis of multimodal data, significantly improving diagnostic accuracy and enhancing the granularity of risk stratification. For instance, a review by Yang et al. highlighted that AI technologies, especially deep learning, have made notable progress in tumor detection, molecular subtyping, staging and grading, prognosis prediction, and recurrence assessment of bladder cancer. Multimodal AI systems enable joint analysis of cystoscopic images, histopathological slides, and molecular biomarker data, thereby enhancing diagnostic efficiency and reproducibility while reducing the risks of misdiagnosis and missed diagnosis (1). In addition, DL models incorporating blood- and urine-based data—such as analyses of complex morphological patterns in dried droplets—have demonstrated noninvasive, highly sensitive detection capabilities, underscoring the clinical promise of multimodal data fusion (26). In the imaging domain, AI diagnostic platforms combined with blue-light cystoscopy have achieved high sensitivity and specificity in assessing tumor malignancy, invasiveness, and grade, further illustrating the clinical value of integrating advanced imaging modalities with AI algorithms (5). Moreover, with the widespread adoption of digital pathology, AI-assisted image analysis enables automated tissue segmentation, histopathological grading, and molecular marker identification, thereby accelerating the development of precision pathological diagnostics (21). The core advantage of multimodal AI systems lies in their ability to leverage complementary information across heterogeneous data types, overcoming the intrinsic limitations of single-modality approaches and enhancing both diagnostic robustness and accuracy. Looking ahead, as data acquisition technologies and algorithmic methodologies continue to evolve, multimodal AI systems are expected to become indispensable auxiliary tools in bladder cancer diagnosis and grading, playing a pivotal role in driving the clinical implementation of precision medicine. To visually summarize the integration of imaging, pathology, and molecular data discussed in this section, a conceptual multimodal AI pipeline for bladder cancer diagnosis and grading is illustrated in Figure 1.

### 6.2 Intelligent decision support systems empowering precision medicine

AI-based intelligent decision support systems can integrate multidimensional and heterogeneous patient data to assist clinicians in making more accurate and evidence-based decisions in real-world clinical settings regarding bladder cancer diagnosis, treatment selection, and prognostic assessment. Precision medicine emphasizes individualized and biology-driven therapy. This paradigm requires in-depth analysis of tumor biology, a need that can be effectively addressed by AI-assisted systems. According to a review by Marra and colleagues on behalf of the European Society for Medical Oncology (ESMO) Precision Medicine Working Group, AI applications in digital pathology have demonstrated improvements in diagnostic accuracy and prognostic evaluation, particularly excelling in automated tumor detection, molecular biomarker identification, and therapeutic response prediction. AI-driven decision support systems enable the integration of multi-omics datasets, facilitating precise patient stratification and the development of personalized therapeutic strategies (21). In clinical pathways, AI technologies are also being applied to assist in radiotherapy planning, treatment response prediction, and evaluation of immunotherapy effectiveness. Hoskin reported that AI has been incorporated into radiotherapy workflows—such as contouring, planning, and quality assurance—substantially enhancing both efficiency and precision (30). In the context of bladder cancer treatment, intelligent decision support systems can recommend the most appropriate therapeutic strategies based on patients’ clinical and molecular characteristics, thereby reducing the risks of overtreatment or undertreatment. The successful implementation of such systems requires close multidisciplinary collaboration among data scientists, clinicians, and engineers to ensure clinical safety and applicability. With the advancement of big data infrastructure and computational power, intelligent decision support systems are expected to play an increasingly pivotal role in the practice of precision medicine for bladder cancer.

### 6.3 Integration of AI with emerging biomarkers

Research into bladder cancer biomarkers has been advancing across multiple levels, including gene expression, proteomics, epigenetics, and nanotechnology. The discovery of emerging biomarkers offers valuable tools for early diagnosis, histopathological grading, and prognostic assessment. The introduction of AI has offered powerful computational support for the interpretation and clinical translation of these complex biomarker datasets. Kim et al. noted that although numerous molecular biomarkers have been proposed for predicting bladder cancer recurrence and progression, the sensitivity and specificity of individual markers remain suboptimal, limiting their standalone clinical utility. The development of high-throughput molecular technologies has rendered gene expression profiling and epigenetic modifications promising comprehensive biomarker candidates. AI, through machine learning algorithms, can extract clinically meaningful feature combinations from large-scale datasets (33). Fan and colleagues further summarized a variety of diagnostic and prognostic biomarkers, including CK, P53, PPAR*γ*, PTEN, and non-coding RNAs, highlighting the potential of AI in integrating multi-biomarker information to address biological heterogeneity. Such integration facilitates more accurate risk stratification and prediction of therapeutic responses (34). In addition, advances in nanomedicine have opened new avenues for the diagnosis and treatment of bladder cancer. Nanoplatforms enable targeted drug delivery and ultrasensitive biomarker detection. When combined with AI-based image recognition and data analytics, these technologies are expected to further improve the noninvasiveness and precision of diagnostic approaches (35). Shi et al. reviewed proteomic, genomic, and transcriptomic analyses based on exosomes, emphasizing the central role of AI in managing these complex datasets and accelerating clinical translation of novel biomarkers (36). Looking forward, the integration of AI with emerging biomarkers holds great promise for early detection, dynamic monitoring, and personalized therapy in bladder cancer, ultimately advancing the goals of precision medicine.

### 6.4 Strategies to promote routine clinical implementation

Despite remarkable progress in the application of AI for bladder cancer diagnosis and grading, its routine clinical implementation still faces multiple challenges, including data heterogeneity, limited model generalizability, insufficient large-scale multicenter validation, and unresolved ethical and regulatory issues. A systematic and coordinated strategy is essential to drive the clinical translation of AI technologies. First, data standardization and the construction of high-quality, well-annotated datasets are fundamental. Gao et al. emphasized that current AI applications in gastrointestinal cancer diagnosis face issues of data heterogeneity and lack of unified standards—challenges that are equally applicable to bladder cancer research. Establishing standardized multicenter databases will substantially enhance the generalizability and clinical applicability of DL models (28). Second, robust clinical validation and regulatory approval are critical. Försch et al. pointed out that although numerous proof-of-concept studies exist, there remains a lack of randomized controlled trials and prospective studies demonstrating tangible clinical benefit. Conducting large-scale, multicenter clinical trials to assess the safety, efficacy, and impact of AI systems on patient outcomes is a necessary step toward clinical adoption (15). At the same time, comprehensive ethical and legal frameworks must be developed and operationalized to ensure patient privacy and data security, address the “black box” problem of AI systems, and build clinician and patient trust. Moreover, interdisciplinary talent development and optimization of clinical workflows are equally important. Goldenberg et al. emphasized that collaboration among clinicians, pathologists, and data scientists is key to the successful implementation of AI technologies. Training healthcare professionals to understand and utilize AI tools, along with optimizing clinical pathways, will ensure the seamless integration of AI-assisted decision-making into routine medical practice (37). In addition to discussing technological advances and implementation challenges, we further analyzed the clinical translation of AI-assisted bladder cancer diagnostics by reviewing ongoing and completed clinical trials. Supplementary Table 1 presents a detailed summary of these trials, including study phases, sample sizes, randomization methods, endpoints, interventions, and trial status. This overview provides a practical reference for understanding the current clinical validation landscape of AI technologies and underscores the need for multicenter, prospective studies to support broader clinical adoption. Finally, policy support and funding are indispensable. A bibliometric analysis by Zhou et al. demonstrated a rapid increase in AI-related bladder cancer research, particularly in the United States and Europe, where supportive policies and sustained funding have accelerated clinical translation (29). In summary, promoting the clinical implementation of AI in bladder cancer diagnosis and grading requires concerted efforts across multiple domains, including data standardization, clinical validation, ethical and legal governance, workforce training, and policy support (38). Only through such multifaceted collaboration can AI truly empower precision medicine.

## 7 Conclusion and outlook

### 7.1 Clinical significance of current research advances

Artificial intelligence has substantially advanced the diagnosis and histopathological grading of bladder cancer, addressing long-standing challenges such as subjectivity and interobserver variability. AI-assisted systems have demonstrated strong performance in tumor detection, grading, staging, and prognostic prediction, offering meaningful support for clinical decision-making. However, these advances should be interpreted with caution. Many reported achievements are derived from controlled research settings and may not fully reflect real-world clinical complexity. The true clinical value of AI lies not only in algorithmic accuracy but also in its reproducibility, interpretability, and integration into routine clinical practice. Recognizing these constraints is essential for preventing overestimation of current capabilities and for guiding responsible clinical adoption.

### 7.2 Future research priorities and potential impact

Future research on AI in bladder cancer must move beyond proof-of-concept studies toward clinically oriented, methodologically rigorous investigations. First, the establishment of large-scale, multicenter datasets with standardized acquisition protocols and high-quality annotations is imperative to enhance model generalizability. Second, improving model interpretability should be treated as a clinical necessity rather than a secondary technical objective, as transparent AI systems are critical for clinician trust, regulatory approval, and ethical accountability. Third, prospective randomized clinical trials are urgently needed to determine whether AI-assisted diagnosis translates into measurable patient benefit to evaluate whether AI-assisted diagnosis meaningfully improves clinical outcomes, workflow efficiency, and healthcare resource utilization. Finally, future AI systems should increasingly adopt multimodal data integration, combining imaging, digital pathology, molecular profiling, and clinical data to better capture the biological complexity of bladder cancer (39). Parallel development of ethical, legal, and regulatory frameworks will be essential to ensure that AI technologies are deployed safely, equitably, and responsibly. Collectively, addressing these priorities will determine whether AI evolves from an experimental tool into a reliable cornerstone of precision medicine for bladder cancer (40).

## References

[1] Yang X Yang R Liu X Chen Z Zheng Q. Recent advances in artificial intelligence for precision diagnosis and treatment of bladder cancer: a review. Ann Surg Oncol. (2025) 32(8):6173–6184. 10.1245/s10434-025-17228-6 40221553

[2] Verma S Rajesh A Prasad SR Gaitonde K Lall CG Mouraviev V Urinary bladder cancer: role of MR imaging. Radiographics. (2012) 32(2):371–87. 10.1148/rg.322115125 22411938

[3] Mitra AP Cote RJ. Molecular pathogenesis and diagnostics of bladder cancer. Annu Rev Pathol. (2009) 4:251–85. 10.1146/annurev.pathol.4.110807.092230 18840072

[4] Ma X Zhang Q He L Liu X Xiao Y Hu J Artificial intelligence application in the diagnosis and treatment of bladder cancer: advance, challenges, and opportunities. Front Oncol. (2024) 14:1487676. 10.3389/fonc.2024.1487676 39575423 PMC11578829

[5] Ali N Bolenz C Todenhöfer T Stenzel A Deetmar P Kriegmair M Deep learning-based classification of blue light cystoscopy imaging during transurethral resection of bladder tumors. Sci Rep. (2021) 11(1):11629. 10.1038/s41598-021-91081-x 34079004 PMC8172542

[6] Wong VK Ganeshan D Jensen CT Devine CE. Imaging and management of bladder cancer. Cancers (Basel). (2021) 13(6):1396. 10.3390/cancers13061396 33808614 PMC8003397

[7] Nafe R Roth S Rathert P. Analysis of criteria for grading bladder cancer in urine cytological tumor diagnosis by means of an expert system. Eur Urol. (1992) 21(2):103–9. 10.1159/000474813 1499608

[8] Akhtar M Al-Bozom IA Ben Gashir M Taha NM. Intrinsic molecular subclassification of urothelial carcinoma of the bladder: are we finally there? Adv Anat Pathol. (2019) 26(4):251–256. 10.1097/PAP.0000000000000235 31188799

[9] Bera K Katz I Madabhushi A. Reimagining T staging through artificial intelligence and machine learning image processing approaches in digital pathology. JCO Clin Cancer Inform. (2020) 4:1039–1050. 10.1200/CCI.20.00110 33166198 PMC7713520

[10] Jiang Y Yang M Wang S Li X Sun Y. Emerging role of deep learning-based artificial intelligence in tumor pathology. Cancer Commun (Lond). (2020) 40(4):154–166. 10.1002/cac2.12012 32277744 PMC7170661

[11] Hafeez S Huddart R. Advances in bladder cancer imaging. BMC Med. (2013) 11:104. 10.1186/1741-7015-11-104 23574966 PMC3635890

[12] Bochenek K Aebisher D Międzybrodzka A Cieślar G Kawczyk-Krupka A. Methods for bladder cancer diagnosis—the role of autofluorescence and photodynamic diagnosis. Photodiagnosis Photodyn Ther. (2019) 27:141–148. 10.1016/j.pdpdt.2019.05.036 31152879

[13] Shkolyar E Zhou SR Carlson CJ Chang S Laurie MA Xing L Optimizing cystoscopy and TURBT: enhanced imaging and artificial intelligence. Nat Rev Urol. (2025) 22(1):46–54. 10.1038/s41585-024-00904-9 38982304 PMC11939114

[14] Liu JJ Droller MJ Liao JC. New optical imaging technologies for bladder cancer: considerations and perspectives. J Urol. (2012) 188(2):361–8. 10.1016/j.juro.2012.03.127 22698620 PMC4035237

[15] Försch S Klauschen F Hufnagl P Roth W. Artificial intelligence in pathology. Dtsch Arztebl Int. (2021) 118(12):194–204. 34024323 10.3238/arztebl.m2021.0011 PMC8278129

[16] Mohammed AA Hani ET El-Khatib HM Mirza AA Mirza AA Alturaifi TH. Urinary bladder cancer: biomarkers and target therapy, new era for more attention. Oncol Rev. (2016) 10(2):320. 28058098 10.4081/oncol.2016.320 PMC5178843

[17] Guo CC Czerniak B. Bladder cancer in the genomic era. Arch Pathol Lab Med. (2019) 143(6):695–704. 10.5858/arpa.2018-0329-RA 30672335

[18] Dyrskjøt L Hansel DE Efstathiou JA Knowles MA Galsky MD Teoh J Bladder cancer. Nat Rev Dis Primers. (2023) 9(1):58. 10.1038/s41572-023-00468-9 37884563 PMC11218610

[19] Kamat AM Hegarty PK Gee JR Clark PE Svatek RS Hegarty N ICUD-EAU international consultation on bladder cancer 2012: screening, diagnosis, and molecular markers. Eur Urol. (2013) 63(1):4–15. 10.1016/j.eururo.2012.09.057 23083902

[20] Bera K Schalper KA Rimm DL Velcheti V Madabhushi A. Artificial intelligence in digital pathology—new tools for diagnosis and precision oncology. Nat Rev Clin Oncol. (2019) 16(11):703–715. 10.1038/s41571-019-0252-y 31399699 PMC6880861

[21] Marra A Morganti S Pareja F Campanella G Bibeau F Fuchs T Artificial intelligence entering the pathology arena in oncology: current applications and future perspectives. Ann Oncol. (2025) 36(7):712–725. 10.1016/j.annonc.2025.03.006 40307127

[22] Sarkar S Min K Ikram W Tatton RW Riaz IB Silva AC Performing automatic identification and staging of urothelial carcinoma in bladder cancer patients using a hybrid deep-machine learning approach. Cancers (Basel). (2023) 15(6):1673. 10.3390/cancers15061673 36980557 PMC10046500

[23] Fuster S Kiraz U Eftestøl T Janssen EAM Engan K. NMGrad: advancing histopathological bladder cancer grading with weakly supervised deep learning. Bioengineering (Basel). (2024) 11(9):909. 10.3390/bioengineering11090909 39329651 PMC11428615

[24] Shmatko A Ghaffari Laleh N Gerstung M Kather JN. Artificial intelligence in histopathology: enhancing cancer research and clinical oncology. Nat Cancer. (2022) 3(9):1026–1038. 10.1038/s43018-022-00436-4 36138135

[25] Tosoni I Wagner U Sauter G Egloff M Knönagel H Alund G Clinical significance of interobserver differences in the staging and grading of superficial bladder cancer. BJU Int. (2000) 85(1):48–53. 10.1046/j.1464-410x.2000.00356.x 10619945

[26] Demir R Koc S Ozturk DG Bilir S Ozata Hİ Williams R Artificial intelligence assisted patient blood and urine droplet pattern analysis for non-invasive and accurate diagnosis of bladder cancer. Sci Rep. (2024) 14(1):2488. 10.1038/s41598-024-52728-7 38291121 PMC10827787

[27] Kiani A Uyumazturk B Rajpurkar P Wang A Gao R Jones E Impact of a deep learning assistant on the histopathologic classification of liver cancer. NPJ Digit Med. (2020) 3:23. 10.1038/s41746-020-0232-8 32140566 PMC7044422

[28] Gao Y Wen P Liu Y Sun Y Qian H Zhang X Application of artificial intelligence in the diagnosis of malignant digestive tract tumors: focusing on opportunities and challenges in endoscopy and pathology. J Transl Med. (2025) 23(1):412. 10.1186/s12967-025-06428-z 40205603 PMC11983949

[29] Zhou Y Xu W Zeng Y Li H Liu Z Wang T Global research trends of the application of artificial intelligence in bladder cancer since the 21st century: a bibliometric analysis. Front Oncol. (2023) 13:1227152. 10.3389/fonc.2023.1227152 38094602 PMC10718619

[30] Hoskin PJ. The use of artificial intelligence technologies in cancer care. Clin Oncol (R Coll Radiol). (2025) 38:103644. 10.1016/j.clon.2024.09.003 39368900

[31] Lai B Fu J Zhang Q Deng N Jiang Q Peng J. Artificial intelligence in cancer pathology: challenge to meet increasing demands of precision medicine. Int J Oncol. (2023) 63(3):1–30. 10.3892/ijo.2023.5555 37539741

[32] Zhang C Xu J Tang R Yang J Wang W Yu X Novel research and future prospects of artificial intelligence in cancer diagnosis and treatment. J Hematol Oncol. (2023) 16(1):114. 10.1186/s13045-023-01514-5 38012673 PMC10680201

[33] Kim WJ Park S Kim YJ. Biomarkers in bladder cancer: present status and perspectives. Biomark Insights. (2007) 2:95–105. 10.1177/117727190700200018 19662195 PMC2717839

[34] Fan J Chen B Luo Q Li J Huang Y Zhu M Potential molecular biomarkers for the diagnosis and prognosis of bladder cancer. Biomed Pharmacother. (2024) 173:116312. 10.1016/j.biopha.2024.116312 38417288

[35] Kong C Zhang S Lei Q Wu S. State-of-the-art advances of nanomedicine for diagnosis and treatment of bladder cancer. Biosensors (Basel). (2022) 12(10):796. 10.3390/bios12100796 36290934 PMC9599190

[36] Shi Y Mathis BJ He Y Yang X. The current progress and future options of multiple therapy and potential biomarkers for muscle-invasive bladder cancer. Biomedicines. (2023) 11(2):539. 10.3390/biomedicines11020539 36831075 PMC9953154

[37] Goldenberg SL Nir G Salcudean SE. A new era: artificial intelligence and machine learning in prostate cancer. Nat Rev Urol. (2019) 16(7):391–403. 10.1038/s41585-019-0193-3 31092914

[38] Yates J Van Allen EM. New horizons at the interface of artificial intelligence and translational cancer research. Cancer Cell. (2025) 43(4):708–727. 10.1016/j.ccell.2025.03.018 40233719 PMC12007700

[39] Zhang Y-H Guo L-J Yuan X-L Hu B. Artificial intelligence-assisted esophageal cancer management: now and future. World J Gastroenterol. (2020) 26(35):5256–5271. 10.3748/wjg.v26.i35.5256 32994686 PMC7504247

[40] Fountzilas E Pearce T Baysal MA Chakraborty A Tsimberidou AM. Convergence of evolving artificial intelligence and machine learning techniques in precision oncology. NPJ Digit Med. (2025) 8(1):75. 10.1038/s41746-025-01471-y 39890986 PMC11785769

